# Sinus Rhythm Propagation and Low-Voltage Bridge in Koch’s Triangle: How They Relate in Cryoablation of Atrioventricular Nodal Reentry Tachycardia in Children

**DOI:** 10.3390/jcm15083058

**Published:** 2026-04-16

**Authors:** Francesco Flore, Michele Lioncino, Pietro Paolo Tamborrino, Ilaria Cazzoli, Alberto Ferraro, Vincenzo Pazzano, Daniele Garozzo, Cristina Raimondo, Massimo Stefano Silvetti, Fabrizio Drago

**Affiliations:** Paediatric Cardiology and Cardiac Arrhythmias Complex Unit, Bambino Gesù Children’s Hospital IRCCS, Piazza Sant’Onofrio, 00165 Rome, Italy; francesco.flore@opbg.net (F.F.); ilaria.cazzoli@opbg.net (I.C.); alberto.ferraro@abbott.com (A.F.); vincenzo.pazzano@opbg.net (V.P.); daniele.garozzo@opbg.net (D.G.); cristina.raimondo@opbg.net (C.R.); mstefano.silvetti@opbg.net (M.S.S.); fabrizio.drago@opbg.net (F.D.)

**Keywords:** atrioventricular nodal reentry tachycardia, pediatric tachyarrhythmias, 3D mapping, low-voltage bridge, sinus propagation mapping, cryoablation

## Abstract

**Background/Objectives**: Transcatheter ablation assisted by three-dimensional (3D) electroanatomical mapping (EAM) is the elective treatment for atrioventricular nodal reentrant tachycardia (AVNRT) in children and adolescents. In this population of patients, the most frequently employed EAM strategies are the low-voltage bridge (LVB) strategy and sinus rhythm propagation mapping (SRPM). However, the exact pathophysiology and anatomy of the AVNRT reentrant circuits are still poorly understood. The aim of this study was to investigate the relationship between SRPM and LVB and to shed light on nodal physiology in children and adolescents affected by AVNRT. **Methods**: We retrospectively collected data on pediatric patients who underwent cryoablation for AVNRT assisted by high-density 3D EAM by using the LVB strategy; maps were reviewed by two independent electrophysiologists and the SRPM was described. SRPM was defined as typical when only one collision area was identified and atypical whenever either no or ≥ two collision areas were localized. **Results**: Twenty-eight consecutive patients (11.3 ± 3.3 years) were enrolled. All procedures were acutely successful. Overall, atypical SRPM was present in 10 patients (35.7%), and it did not correlate with the presence of multiple SPs or electrophysiological data. Moreover, we observed an imperfect concordance between SRPM and LVB (only in 10/18 patients). When SRPM and LVB were assessed in different locations, the LVB identified the effective cryoablation site in more cases than SRPM (4/8 vs. 1/8). Lastly, in cases of double collision, one collision area co-localized with the LVB and the effective cryoablation spot, whereas the other was located superiorly, closer to the His bundle. **Conclusions**: Atypical sinus rhythm propagation in the Koch’s triangle is a frequent finding in pediatric AVNRT patients. In this series, LVB showed closer concordance with the successful cryolesion site than retrospectively reconstructed SRPM.

## 1. Introduction

Atrioventricular nodal reentrant tachycardia (AVNRT) is a regular, narrow complex QRS tachycardia, accounting for 13–34% of supraventricular tachycardias (SVTs) in children and adolescents. The underlying arrhythmic mechanism involves nodal conduction over a so-called “slow pathway” and reentry over another slow pathway or the normal fast pathway, to produce a reentrant tachycardia. Older patients usually present with palpitations and/or chest pain, either at rest or during exercise, and more rarely dizziness and/or syncope; infants may exhibit signs of poor feeding, profuse sweating with feeding, and/or fast breathing, and/or they can appear generally ill. Unfortunately, AVNRT episodes usually become more frequent with growth, requiring pharmacological treatment or transcatheter ablation [1]. At present, transcatheter ablation of the slow pathway (SP) is the preferred treatment for AVNRT in pediatric patients, especially if they are >5 years old or >20 kg [1,2].

In this particular setting, three-dimensional (3D) electroanatomical mapping (EAM) systems seem to enhance procedural success and minimize complication rates, especially when the low-voltage bridge (LVB) strategy or sinus rhythm propagation mapping (SRPM) is used [3,4,5,6].

The LVB involves direct visualization of the slow pathway (SP) using voltage gradient mapping of Koch’s triangle (KT), whilst SRPM involves the propagation mapping of the sinus impulse in the right atrium, which, in AVNRT patients, seems to be peculiar due to the presence of fast and slow pathways. Identification of the “wave collision area” (WCA) or the “pivot point” (PP) is used to locate the correct ablation point for the SP [5,7,8,9].

Both strategies seem to be very effective, but data comparing the results of these mapping strategies is reported in the literature in only two studies that include less than 50 patients in total [9,10].

Therefore, the aim of this study was to compare the above-mentioned strategies in targeting ablation and to shed light on nodal physiology in children and adolescents with AVNRT.

## 2. Materials and Methods

In this retrospective study, all consecutive pediatric patients with AVNRT who underwent high-density 3D transcatheter cryoablation at our institution from October 2024 to February 2025 were enrolled. According to the current guidelines, patients underwent transcatheter ablation due to recurrent symptoms [2,11].

Written informed consent was obtained from all patients’ parents prior to the procedure.

### 2.1. Three-Dimensional Electroanatomical Mapping (EAM) and Electrophysiological Study (EPS)

All procedures were performed under general anesthesia, induced with sevoflurane or propofol and maintained with sevoflurane. A thermal mattress was used to maintain normal body temperature. All antiarrhythmic drugs were discontinued for at least five half-lives before the procedure to ensure complete pharmacological washout.

Surface electrocardiogram (ECG) leads and endocardial potentials (EGMs) were recorded and stored on a multichannel recorder (Bard Electrophysiology, Billerica, MA, USA). The bipolar bandwidth filter was set at a range of 30–300 Hz.

Programmed stimulation with atrial or ventricular single, double, and triple premature extrastimuli, as well as incremental pacing and overdrive pacing, was used to induce and confirm the diagnosis of AVNRT according to the EHRA and AEPC guidelines. The same stimulation protocol was repeated under isoproterenol infusion (0.04–0.08 μg/kg/min in incremental doses) if tachycardia was not inducible at baseline.

In detail, the diagnosis of AVNRT was established in cases of paroxysmal supraventricular tachycardias with V = A, VA < 70 ms, concentric atrial activation (in typical forms), and one or more of the following findings: 1. “AH response” to ventricular overdrive pacing; 2. post-pacing interval minus tachycardia cycle length (PPI − TCL) following entrainment from the RV apex of more than 115 ms; 3. absolute “preexcitation index” greater than 100 ms; and/or 4. His refractory premature ventricular extrastimulus resulting in no change in the A-A cycle length.

An atrial–His (AH) jump, diagnostic of dual AV nodal physiology, was defined as a sudden prolongation of the AH interval by 50 ms or greater following a shortening of the paced cycle length or of the coupling interval of the atrial extrastimulus by 10 ms.

The EnSite Precision™ system EE3000 v.2.0 (Abbott Medical Italia SRL, Sesto San Giovanni, Milano, Italy) 3D mapping system was used in all procedures. The Advisor HD Grid™ was used for high-density 3D mapping. All the maps were set with the same values of interpolation, interior, and exterior projection at 7 mm.

Catheters were introduced inside the right atrium from the femoral vein access site to create peak-to-peak voltage maps in sinus rhythm to find SP-associated LVBs, as described in detail elsewhere [1,6]. Briefly, we created a high-density voltage map for the interatrial septum (IAS) within the KT. The voltage color bar was then individually adjusted to highlight an LVB, if present, starting from a voltage range of 0.1–2 mV and then reduced as much as possible. Simultaneously, the SP potential (also called “double potential”, “Jackman potential”, or “hump and spike potential”) was searched and its position analyzed in relation to the area of the LVB (the so-called “electrophysiologically guided LVB strategy”). LVBs were classified, as already reported, as type 1 and type 2. A type 1 LVB is a clear, large area of low voltage between the CS ostium and the AV node with the base on the edge of the tricuspid annulus; a type 2 LVB is a narrower and smaller area of low voltage between adjacent normal-voltage regions in the same area [1].

After the cryoablation procedure, maps were reviewed by two independent electrophysiologists and the SRPM was described. They were blinded to the procedural outcomes or effective ablation sites. In case of disagreement, a third electrophysiologist was consulted. For this purpose, EGMs were used to generate and interpret sinus rhythm activation vectors and LAT maps. The WCA was defined as the area in the KT where the two wavefronts of the sinus impulse meet each other (one traveling in a superior–inferior direction—via the fast pathway—and the other in an inferior–superior direction—via the SP). The PP was defined as the area, within the KT, of a marked change in activation direction of the sinus impulse. SRPM was defined as typical when only one WCA/PP was identified and atypical whenever either no or ≥ two WCAs/PPs were localized.

WCAs and PPs were combined into a single analytical category as they both represent a visualization of conduction over the SP on sinus rhythm propagation maps.

### 2.2. Cryoablation Procedure

The cryoablation system consists of a central console (Cryo Console, CryoCath Technologies Inc., Montreal, QC, Canada) and a steerable 7 Fr catheter (Freezor, CryoCath Technologies Inc., Montreal, Canada) using N2O as a refrigerant. The cryoablation catheter was advanced to the site of interest from the right femoral vein. A 4 or 6 mm tip electrode was preferably used in patients <25 or >25 kg, respectively.

The cryoablation procedure was performed as previously described in detail elsewhere [1]. Briefly, during cryomapping, repeat extrastimulus testing or ramps were performed using the diagnostic catheter placed in the high right atrium to assess tachycardia inducibility or the absence of AV nodal SP conduction. If cryomapping was effective (i.e., no AH jump, no reentry beats, and no inducibility of tachyarrhythmia), the tip temperature was further reduced to create a permanent lesion. In contrast, if cryomapping produced unwanted effects (e.g., transient high-degree AV block, lengthening of the PR interval), cryoapplication was discontinued to allow tissue rewarming and reversibility of electrical function loss.

In order to consolidate the lesion, after a successful cryoablation, extra lesions (“cryo-bonuses”) following the initially successful lesion were placed at the site and immediately adjacent to the site on both sides and on the ventricular and atrial aspect of the tricuspid annulus, creating eventually different types of lesion, namely the focal lesion, the extended focal lesion, the “high-density linear lesion” (HDLL) and the extended HDLL, according to patients’ anatomical substrate.

In all patients, a post-ablation electrophysiological study at baseline and during isoproterenol infusion was performed immediately and after 30 min to demonstrate complete and persistent interruption of retrograde and anterograde conduction over the AP and/or non-inducibility of AVRT.

An acutely successful procedure was defined by the non-inducibility of AVNRT and the absence of spontaneous recurrences in the first 24 h of observation.

### 2.3. Post-Ablation Assessment and Follow-Up

After the procedure, all patients were carefully monitored. A standard ECG was performed 24 h post-procedure in all patients.

Subsequent follow-up included a cardiac examination and an ECG performed one month after the procedure. Moreover, patients were encouraged to seek care at the local emergency room in case of symptoms similar to those prior to the ablation.

Early recurrence was defined as ECG-documented tachycardia or the return of clinical symptoms identical to those before cryoablation during this period of time.

As a safety endpoint, transient and permanent complications were considered.

### 2.4. Statistical Analysis

All continuous variables were assessed for normality with a one-sample Kolmogorov–Smirnov test and by examination of their histogram. When normal distribution was identified, variables were expressed as means and standard deviations (SDs) and tested for differences using Student’s unpaired *t*-test. Non-parametric variables were expressed as the median and interquartile range (IQR), and differences were tested using the Mann–Whitney test, as appropriate. Categorical variables were expressed as percentages and analyzed using Fisher’s exact test. All statistical tests were two-sided, and a *p*-value < 0.05 was considered statistically significant. All statistical analyses were performed using MedCalc Statistical Software version 15.8 (MedCalc Software bvba, Ostend, Belgium; https://www.medcalc.org; 2015).

## 3. Results

### 3.1. Characteristics of the Study Population

There were 28 children in the study (42.8% males; mean age 11.3 ± 3.3 years; mean weight 47 ± 15.6 kg). All of them had a prior diagnosis of AVNRT, either through ECG or through transesophageal atrial pacing (TAP).

Eleven (39.3%) were on antiarrhythmic drugs before the ablation procedure (nine on flecainide; two on beta-blockers).

### 3.2. Electrophysiological and Cryoablation Data

During EPS, 23 patients (82.1%) showed inducibility of AVNRT, either at rest or during isoproterenol infusion; typical AVNRT was evident in 24 patients (85.7%) and atypical AVNRT in 4 (14.3%).

When considering both the ablation procedure and the previous TAP procedure, an AV nodal (AH) “jump” was present in 18/28 (64.2%), while the presence of Stimulus-R > RR was observed in 14/28 (50%). During the ablation procedure, under general anesthesia, an AV nodal (AH) “jump” was present in 14/28 (50%), while the presence of Stimulus-R > RR was observed in 13/20 (65%).

All patients underwent an acutely successful cryoablation.

The ablation catheter used was a 6 mm tip, 7 Fr cryoablation catheter (Freezor, Medtronic Cryocath LP, Montreal, Quebec, Canada) in all patients but two, for whom a 4 mm tip cryoablation catheter was used (all <25 kg of weight). In one patient, a 6 mm tip catheter was subsequently substituted with an 8 mm tip catheter.

With regard to the effectiveness of the ablation in terms of AVNRT non-inducibility, the median effective cryoablation attempt was the first (IQR 1–4). The total number of cryoapplications per procedure was a median of 4 (IQR 3.5–6).

No major or minor adverse events were observed during the procedure and over the following in-ward follow-up pre-discharge.

General, electrophysiological, and cryoablation details of the study are included in Table 1.

### 3.3. Three-Dimensional Mapping Data

Three-dimensional voltage and propagation mapping of IAS and KT was conducted in all patients, with a median of 2365 ± 1640 points.

LVBs were detected with a voltage ranging from 0.2± 0.16 to 1.2± 0.8 mV. One LVB was found in all patients but three, among whom two LVBs were found. A type 1 LVB was found in 11 patients (39.3%), and a type 2 LVB in 17 patients (60.7%).

**Figure 1:**
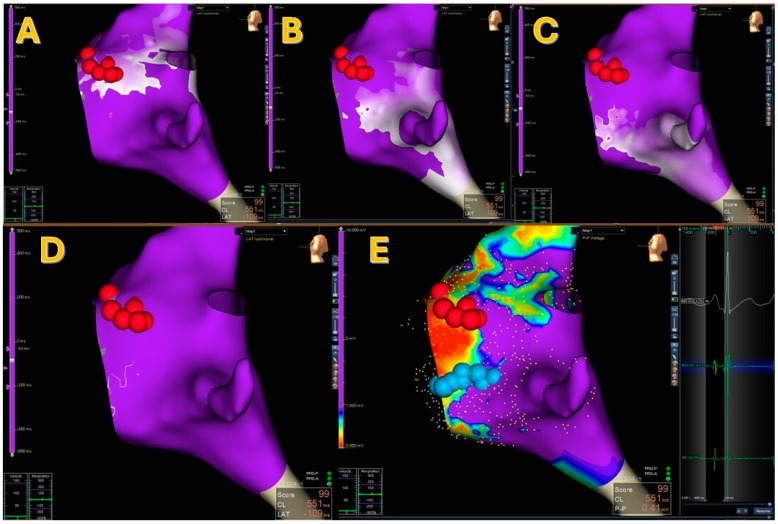
A case of SRPM showing a pivot point in the mid-posterior septum (Panels **A**–**D**) and its relationship with the LVB and the cryoablations (Panel **E**). Red dots indicate His locations, blue dots indicate cryoablation areas.

**Figure 2:**
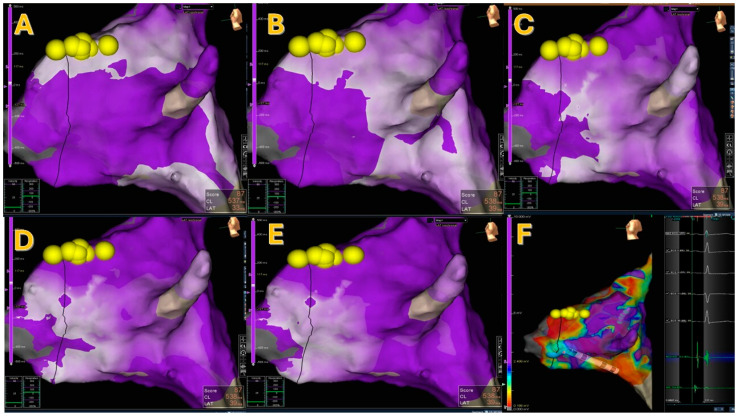
A case of atypical SRPM showing two areas of wave collision, one in the mid-septum and the other in the posterior septum (Panels **A**–**E**): Panel **F** shows a type 1 LVB with “hump and spike” signals and the cryoablations. Yellow dots indicate His locations, blue dots indicate cryoablation spots.

**Figure 3:**
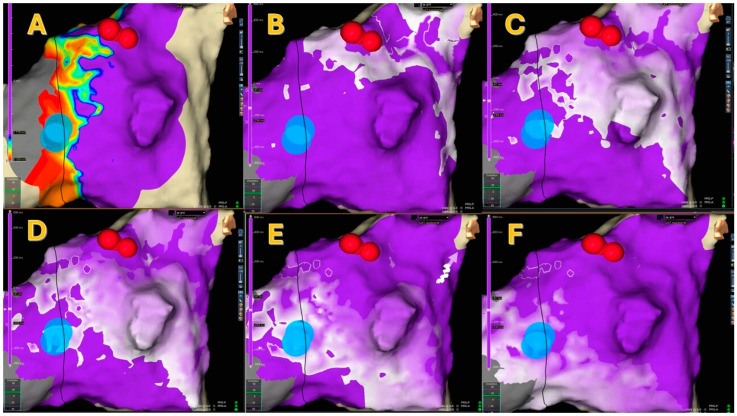
Panel A shows a type 2 LVB in the mid-posterior septum and the effective cryoablations. In this patient, SRPM (Panels B–F) did not show either a pivot point or a wave collision. Red dots indicate His locations, blue dots indicate cryoablation areas.

After reviewing the maps post-processing, SRPM showed the presence of a PP in 4 patients (14.3%; see Figure 1 and Supplemental Video S1), a WCA in 14 (50%), two or more WCAs in 4 (14.3%; see Figure 2), and neither a PP nor a WCA in 6 (21.4%; see Figure 3).

The mean number of WCAs/PPs was 1 ± 0.7, while the number of LVBs was 1.1 ± 0.3 (*p* = 0.29). In two out of three patients with two LVBs, more than one WCA was observed.

Typical SRPM was present in 18/28 (64.3%), whilst atypical SRPM was present in 10/28 (35.7%) patients.

The presence of atypical SRPM did not correlate with specific electrophysiological parameters and the presence of atypical AVNRT (see Table 2). In three out of the four patients with ≥ two WCAs/PPs, one of the WCAs/PPs co-localized with the LVB and the effective cryoablation spot, while the other WCA/PP was located superiorly (see Figure 2). Only in one patient was the effective cryoablation spot located between the WCAs (both superior) and the part of the LVB with SP potentials (inferior).

In cases of typical SRPM, WCAs/PPs and the LVB co-localized in 10/18 (55%) patients, corresponding as well to the effective cryoablation spot (see Figure 1). When SRPM and LVB were carried out in different locations, the LVB co-localized with the site of successful cryoablation in a greater number of cases (4/8 vs. 1/8), even though it did not reach a statistical difference (*p* = 0.12). In addition, the mean difference between the LVB and the effective cryoablation spot vs. WCA/PP and the effective cryoablation spot was 1.09 mm ±1.4 mm vs. 2.3 mm ±2.3 mm (*p* = 0.0572).

### 3.4. Three-Dimensional Mapping Data in Patients with Atypical AVNRT

Among the four patients with atypical AVNRT, one had two LVBs, one had a type 2 LVB, and two had a type 1 LVB. None presented with an atypical SRPM; in three, a single WCA was found, whilst only a single PP was found in the remainder.

With regard to the effective cryoablation spot, it was located between the main LVB and the WCA in the patient with two LVBs (this was the only case in the entire cohort in which neither the LVB nor SRPM predicted precisely the correct ablation site); among the remaining three patients, in one case, it co-localized with both the LVB and SRPM, whereas in two cases, it was predicted by the LVB but not SRPM.

## 4. Discussion

The 3D voltage gradient mapping of KT in AVNRT patients has been used over the last 10 years to allow SP localization. First described in the adult population, it was later adopted by pediatric electrophysiologists as well [3,4,6,12,13].

With the introduction of the LVB concept, ablation results improved in terms of both efficacy (success rate increased from 96 to 100% and recurrence rate from 0 to 7%, also with cryoenergy) and safety (no reported cases of permanent AV nodal block) [1].

More recently, another strategy has been described to localize the SP: the so-called SRPM. However, most data on this mapping strategy derive from adults, and only a few pediatric studies have explored its application in this population [7,8,14,15].

In this study, we analyzed data from AVNRT ablation procedures in children and adolescents for which the LVB strategy was used, and we re-analyzed the maps to look for sinus rhythm propagation. As far as we know, this is the first paper to directly describe atypical patterns in SRPM in pediatric patients and to illustrate their relationship with the LVB.

As a preliminary relevant observation, an acutely successful ablation was achieved in all patients included in this study, confirming the efficacy of the “electrophysiologically guided” LVB strategy in the treatment of AVNRT in children [6].

Interestingly, atypical SRPM did not correlate with specific electrophysiological parameters or with the presence of atypical AVNRT in our population. In these few cases, the voltage gradient maps also did not show multiple LVBs consistently, suggesting that both mapping strategies can fail in identifying SPs different from the right inferior extension of the AV node and that other strategies may be needed [16,17].

However, due to the limited number in this single-center population, this study cannot draw definite conclusions regarding the relationship between atypical SRPM, atypical AVNRT, and the presence or absence of multiple slow pathways.

Another important finding is that, when two or more WCAs/PPs were present, the inferior one co-localized with the LVB and the effective cryoablation spot, whereas the other one was closer to the His bundle, possibly misleading the operator. Consequently, it is possible that reliance on SRPM alone may be problematic in some pediatric cases.

Even though the small number of patients included in this retrospective series does not allow us to draw definite conclusions, our findings could be speculatively explained by the following reasons:

1. Propagation maps depend on the functional refractory periods of the structures involved at the moment of the registration. In this regard, it must be remembered that in our population, like in most pediatric centers, ablations were performed under general anesthesia, which can modify atrial, transitional cells and AV nodal conduction times and, consequently, the appearance of a propagation map.
2. AV nodal structures (including AV nodal extensions) undergo evolutive changes during growth; thus, it might be possible that AVNRT circuits and substrates are slightly different in pediatric populations.
3. The use of HD mapping in our cohort may have helped identify these variants of SRPM.

In this study, we also found that in more than half of the patients, the LVB and SRPM are concordant, and in all these cases, they co-localize with the correct ablation site. However, in our series, when their locations differed, the LVB showed closer concordance with the successful cryolesion site than retrospectively reconstructed SRPM.

In this regard, the available pediatric literature shows inconsistent results.

Van Aartsen et al. [7] reported that the successful ablation spot was within the low-voltage area of the KT, but a few millimeters superior to the collision site. Unlike their observations, in our cohort, the WCA/PP was more frequently located superiorly to the effective cryoablation site, especially in cases of atypical SRPM, highlighting the variability of propagation maps in AVNRT patients.

Similarly to our findings, Bailin demonstrated that PPs co-localized with both low-amplitude, fractional EGMs within the TK and with sites of successful SP modification [5].

O’Leary et al., using only HD mapping catheters, reported the PP as the most efficient way to detect the successful ablation site, and stated that bipolar LVBs were observed in only 38% of AVNRT patients. However, the authors used only fixed-voltage cutoffs and did not search for “SP potentials”, which is a major confounding factor. Moreover, results from this study may have been biased by the very low number of patients included [9].

On the other hand, in a recent study by Tseng et al. [10], the authors reported the possible presence of multiple WCAs. In these circumstances, in the study, all points were marked and progressively targeted from the furthest to the closest to the His region. However, while in three patients there was also electrophysiological evidence of multiple SPs and targeting all collision points resulted in procedure success, in the other two patients, multiple WCAs were not associated with multiple SPs, confirming the lack of specificity of this finding, as in our work. Additionally, in the same study, in approximately 30% of patients, either no LVB was found, or it did not correspond to the final successful ablation site. Differently, in our study, the LVB, which was always visible, more frequently co-localized with the correct ablation site. It is possible that this difference relies on the fact that both mapping techniques are operator-dependent and require an “idealization” of the map; in our group, the vast experience with the LVB strategy could have influenced the results.

Overall, our study sheds light on possible different scenarios for AVNRT mapping, possibly related to different anatomical substrates in children. This is extremely important to acknowledge, as the use of SRPM might be confounding in some cases and lead electrophysiologists to ablate closer to the AV node and the fast pathway when it is not necessary.

Furthermore, even in those cases in which multiple WCAs are associated with multiple SPs, it is possible that one of them is not implicated in the AVNRT circuit in that specific patient. Indeed, SP modulation rather than elimination is commonly associated with comparable long-term results, and a more conservative ablation strategy in children may be advisable. Indeed, in AVNRT patients undergoing ablation, the risk of late pacemaker implantation after AVNRT ablation is significantly higher than that in the general population (even higher than the risk of acute, periprocedural pacemaker implantation) [18,19].

### Limitations of the Study

A few limitations must be acknowledged.

First, this is a retrospective, single-tertiary-center study, involving a relatively limited number of patients; therefore, selection bias is possible. Since the sample size was determined by data availability and not a priori power calculation, it is not possible to ascertain whether the study is adequately powered to detect significant differences or associations.

Moreover, the center’s experience with the LVB strategy and the cryoablation could have influenced the results. In addition, cryoablations were performed based on the “electrophysiologically guided LVB strategy”; therefore, comparisons between the LVB and SRPM are biased by the ablation strategy. In other words, this was not a true head-to-head comparison of two mapping strategies, and “structurally”, the study was predisposed to favor LVB because the procedural endpoint was reached using that strategy.

Furthermore, the small number of patients could have affected the statistical relevance of some results.

Since the follow-up data is limited, this study can only support conclusions regarding acute localization and procedural concordance between the two mapping systems, not durable efficacy.

Another limitation of our study is that we used only one mapping system for EAM-guided cryoablation of AVNRT, which might have yielded different results compared to other mapping systems or radiofrequency energy.

Thus, larger randomized multicenter population studies with fixed protocols are advocated to accurately establish the effectiveness of different mapping strategies and to better understand AVNRT circuits in pediatric patients.

## 5. Conclusions

The strategic combination of the electrophysiologically guided LVB strategy and SRPM can be a valuable solution in cryoablation of AVNRT in children and adolescents. However, sinus rhythm propagation in the KT can be atypical in pediatric AVNRT patients. In this series, LVB depiction on EAM showed closer concordance with the successful cryolesion site than retrospectively reconstructed SRPM when the two were discordant. Consequently, reliance on SRPM alone may be problematic in some pediatric cases.

## Figures and Tables

**Table 1:** Electrophysiological and cryoablation data of the study population.

	Patients (n 28)
**General information**	
Males	12/28 (42.8%)
Age (y)	11.3 ± 3.3
Weight (kg)	47 ± 15.6
Mild CHD	4/28 (14%)
Patients on antiarrhythmic drugs before ablation	11/28 (39.3%)
- Class Ic	9/11
- Beta-blockers	2/11
**Procedural data**	
Fluoroscopy time (min)	0 (IQR 0–0)
Procedural time (min)	203 ± 42.4
Complications	0/28
Acute success	28/28 (100%)
Early recurrences	0/0
Inducible AVNRT at ablation time	23/28 (82.1%)
Atypical AVNRT	4/28 (14.3%)
Tachycardia cycle length (msec)	305 ± 75
VA interval (msec)	45 ± 19.8
AV nodal “jump”	18/28 (64.2%)
Stimulus-R > RR	14/28 (50%)
ERP FP (msec)	325 ± 58
ERP SP (msec)	238 ± 42
WCL (msec)	282 ± 60
**Mapping data**	
LVB	
- Type 1	11/28 (39.3%)
- Type 2	17/28 (60.7%)
Number of LVBs per patient	1.1 ± 0.3
Type of SRPM	
- Wave collision	17/28 (60.7%)
- Pivot point	5/28 (17.9%)
Number of collision areas/pivot points	1 ± 0.7
Atypical sinus propagation	10/28 (35.7%)
Co-existence of LVB and wave collision/pivot point in typical SRPM	10/18 (55%)
In patients without co-existence of LVB and SRPM	
- Co-localization of LVB and effective cryoablation spot	4/8 (50%)
- Co-localization of SRPM and effective cryoablation spot	1/8 (12.5%)

**Table legend.** AV: atrioventricular; AVNRT: atrioventricular nodal reentrant tachycardia; CHD: congenital heart disease; ERP: effective refractory period; FP: fast pathway; LVB: low-voltage bridge; SP: slow pathway; SRPM: sinus rhythm propagation mapping; VA: ventriculo-atrial; WCL: Wenckebach cycle length.

**Table 2:** Differences between typical and atypical SRPM.

	Typical SRPM (18)	Atypical SRPM (10)	*p*-Value
AV nodal “Jump”	12/18 (66.7%)	6/10 (60%)	0.99
Stimulus-R > RR	9/13 (69%)	5/8 (62.5%)	0.99
Tachycardia cycle length (msec)	289 ± 58	295 ± 7	0.89
VA interval (msec)	41 ± 14	46 ± 19	0.65
WCL	270 ± 55	240 ± 56	0.48
Atypical AVNRT	4/16 (25%)	0/8 (0%)	0.26
More than one LVB per patient	1/18 (5.6%)	2/10 (20%)	0.28
Type 2 LVB	10/18 (55.6%)	7/10 (70%)	0.69
Median fluoroscopy time (s)	0	0	0.63
Procedure duration (min)	202 ± 33	226 ± 73	0.56
Number of cryoapplications per procedure	4 (3.5–6)	4.5	0.95

**Table legend.** AVNRT: atrioventricular nodal reentrant tachycardia; SRPM: sinus rhythm propagation mapping; VA: ventriculo-atrial; WCL: Wenckebach cycle length.

## Data Availability

The data underlying this article will be shared on reasonable request to the corresponding author.

## Supplementary Materials

The following supporting information can be downloaded at https://www.mdpi.com/article/10.3390/jcm15083058/s1, Video S1: An example of sinus rhythm propagation mapping.

## Abbreviations

The following abbreviations are used in this manuscript:

3D EAM	three-dimensional electroanatomical mapping
AVNRT	atrioventricular nodal reentrant tachycardia
CHD	congenital heart disease
ECG	electrocardiogram leads
EGM	endocardial potentials
ERP	effective refractory period
EPS	electrophysiological study
FP	fast pathway
HDLL	high-density linear lesion
IAS	interatrial septum
KT	Koch’s triangle
LVB	low-voltage bridge
PP	pivot point
SP	slow pathway
SRPM	sinus rhythm propagation mapping
TAP	transesophageal atrial pacing
WCA	wave collision area
WCL	Wenckebach cycle length
